# Liver resection surgery versus thermal ablation for colorectal LiVer MetAstases (LAVA): study protocol for a randomised controlled trial

**DOI:** 10.1186/s13063-018-2499-5

**Published:** 2018-02-13

**Authors:** Kurinchi Gurusamy, Neil Corrigan, Julie Croft, Maureen Twiddy, Stephen Morris, Nick Woodward, Steve Bandula, Daniel Hochhauser, Vicky Napp, Alison Pullan, Nicholas Jakowiw, Raj Prasad, Steven Olde Damink, C. J. H. M. van Laarhoven, Johannes H. W. de Wilt, Julia Brown, Brian R. Davidson

**Affiliations:** 1 Royal Free Campus, Division of Surgery and Interventional Science, University College London, 9th Floor, Royal Free Hospital, Rowland Hill Street, NW3 2PF, London, UK; 2 Clinical Trials Research Unit, Leeds Institute of Clinical Trials Research Unit, University of Leeds, Leeds, UK; 3 Institute of Clinical and Applied Health Research, University of Hull, Hull, UK; 4 Department of Applied Health Research, University College London, London, UK; 5 Department of Radiology, Royal Free Hospital, London, UK; 6 Department of Radiology, University College London Hospital, London, UK; 7 Cancer Institute, University College London, London, UK; 8 Department of Surgery and Transplantation, Leeds Teaching Hospital, Leeds, UK; 9 Department of General Surgery, Maastricht University, Maastricht, The Netherlands; 10 Department of Surgery, Radboud University Medical Center, Radboud, The Netherlands

**Keywords:** Randomised controlled trial, Cost-benefit analysis, Liver, Neoplasm metastasis, Colorectal neoplasms, Hepatectomy, Ablation techniques

## Abstract

**Background:**

Although surgical resection has been considered the only curative option for colorectal liver metastases (CLM), thermal ablation has recently been suggested as an alternative curative treatment. A prospective randomised trial is required to define the efficacy of resection vs ablation for the treatment of colorectal liver metastases.

**Methods:**

*Design and setting:* This is a multicentre, open, randomised controlled non-inferiority trial design with internal pilot and will be performed in tertiary liver centres in UK and The Netherlands.

*Participants:* Eligible patients will be those with colorectal liver metastases at high surgical risk because of their age, co-morbidities or tumour burden and who would be suitable for liver resection or thermal ablation.

*Intervention:* Thermal ablation as per local policy.

*Control:* Surgical liver resection performed as per centre protocol.

*Co-interventions:* Further chemotherapy will be offered to patients as per current practice.

*Outcomes*

Pilot study: Same as main study and in addition patients and clinicians’ acceptability of the trial to assist in optimisation of recruitment.

Primary outcome: Disease-free survival (DFS) at two years post randomisation.

Secondary outcomes: Overall survival, timing and site of recurrence, additional therapy after treatment failure, quality of life, complications, length of hospital stay, costs, trial acceptability, DFS measured from end of intervention.

*Follow-up:* 24 months from randomisation; five-year follow-up for overall survival.

*Sample size:* 330 patients to demonstrate non-inferiority of thermal ablation.

**Discussion:**

This trial will determine the effectiveness and cost-effectiveness of thermal ablation vs surgical resection for high-risk people with colorectal liver metastases, and guide the optimal treatment for these patients.

**Trial registration:**

ISRCTN Registry, ISRCTN52040363. Registered on 9 March 2016.

**Electronic supplementary material:**

The online version of this article (10.1186/s13063-018-2499-5) contains supplementary material, which is available to authorized users.

## Background

### Colorectal liver metastases

Bowel cancer (colorectal cancer) is the UK’s second biggest cancer killer and the fourth most common cancer. Over 41,000 people are diagnosed with bowel cancer each year in the UK and about 34,000 in England alone. Just under 16,000 colorectal cancer patients die each year in the UK, equating to one death every 32 min [1, 2]. About 20% of patients have liver metastases at presentation [3] and another 30% subsequently develop liver metastases [4]. The resection of colorectal liver metastases (CLM) has provided a good long-term survival for many patients who would have previously been treated with palliative therapy alone [5–8]. However, only about 7–20% of people with CLM undergo potentially curative liver resection because of the age and co-morbidities of the patient or because of the extent of cancer spread [7]. Increasing the number of patients who can undergo potentially curative therapy for liver metastases alone is a main NHS goal for improving the outcome for bowel cancer patients in the UK. Therefore, specialist liver resection centres are carrying out more extensive and complex resections including elderly patients with major co-morbidity. This more extensive surgery in patients with co-morbidity is associated with an increased morbidity and mortality (high-risk patients) [9–14].

### Thermal ablation

Thermal ablation is an alternative modality for treatment of CLM and involves destruction of cancer by heat. Thermal ablation includes established modalities such as radiofrequency ablation (RFA) or microwave ablation (MWA).

#### Radiofrequency ablation

RFA involves localised destruction of the tumour using heat generated by high-frequency alternating current to produce coagulative necrosis of the tumour [15]. For the treatment of CLM, it is generally carried out as a short-stay procedure under general anaesthesia, although it can also be performed under local anaesthesia in some patients [15]. Multiple sessions may be required to treat all the tumours in some patients. It can be performed percutaneously under image guidance (usually CT scan) but can also be performed by open or laparoscopic surgery [15]. Contraindications for RFA include lesions close to the hepatic hilum or adjacent to the hepatic duct as injury may lead to delayed stenosis of the duct and lesions abutting the bowel because of the risk of perforation [15]. Lesions near large blood vessels are often difficult to treat because of dissipation of heat by circulation [15]. According to the Cardiovascular and Interventional Radiological Society of Europe (CIRSE) criteria, RFA is recommended for CLM only when there are ≤ 5 lesions and tumour size does not exceed 3 cm at its longest axis [16]. The dose delivered for RFA varies from one patient to another and is guided by the ablation zone diameter. The target is to heat the tissue to 60 °C at which coagulative necrosis occurs but keep the electrode tip temperature < 100 °C to avoid charring and vaporisation of tissue [15].

#### Microwave ablation

MWA involves localised destruction of the tumour using heat generated by microwave [17–19]. For the treatment of liver lesions, it is usually carried out as a short-stay procedure under general anaesthesia as with RFA, although it can also be performed under local anaesthesia in some patients [17]. As with RFA, multiple sessions may be required in some patients to treat all the lesions (index disease). It is usually performed percutaneously under image guidance (usually ultrasound scan) [17]. It is more effective than RFA in lesions near large blood vessels [17–19]. Major technical limitations of MWA include low power, shaft heating, large diameter probes, long and relatively thin (1–2 cm) ablation zones, and unpredictability regarding the size and shape of the zone of ablation [17].

#### Effectiveness of ablation methods

These methods are currently used for patients with CLM not suitable for surgical resection [20] and not for patients with a possibility of curative liver resection surgery because of high local recurrence rates with thermal ablation [21]. Multiple studies have highlighted the superiority of surgery to ablation for preventing recurrence within the liver [21, 22]. A recent series from Nishiwada et al. showed a 13% recurrence after surgery as opposed to 46% after thermal ablation [21]. Other newer modalities of thermal ablation include laser ablation and high-intensity focused ultrasound (HIFU) [23, 24]. Thermal ablation may be associated with a lower chance of cure than surgery because of the problem with local recurrence. To determine the evidence for thermal ablation, a National Institute for Health Research (NIHR) Health Technology Assessment (HTA)-funded systematic review of literature was commissioned and subsequently published in February 2014 [25]. The systematic review identified one non-randomised comparative study in which the survival in patients with RFA was similar to liver resection surgery despite the RFA group having more co-morbidities or more extensive liver metastases [26]. An exploratory cost-effectiveness analysis performed by the group based on this non-randomised study showed that RFA has the potential to be cheaper and might result in better health-related quality of life. Another non-randomised study published since this systematic review has also shown that patients undergoing RFA have survival comparable to surgery despite having more extensive liver metastases [22]. Similarly, an underpowered randomised controlled trial (RCT) showed no evidence of difference in survival between MWA and liver surgery in resectable CLM [27]. However, in another non-randomised study published after the systematic review by Loveman et al., people who were eligible for surgery but preferred RFA had poorer survival than those undergoing surgery [28].

#### Comparison of methods of thermal ablation

Non-randomised studies comparing MWA with RFA suggested that MWA is better than RFA in terms of technical feasibility and lower disease recurrence in patients with unresectable CLM [25]. Of the newer forms of ablative methods, such as HIFU, CyberKnife®, and cryotherapy, there are no publications comparing these newer ablation methods with either RFA or MWA. In reality, different surgeons and radiologists have their own preferences of method of ablation because of this uncertainty.

### Potential advantages of thermal ablation over surgery

Current evidence suggests that thermal ablation has lower complication rates and better health-related quality of life than surgery [25, 29]. Thermal ablation is also less expensive than liver resection [25], which will result in cost savings to NHS. Ablation therapy has the potential to decrease the pain after treatment and time taken for recovery from cancer therapy which will decrease the number of work days lost by the patient and relatives.

### Potential concerns about thermal ablation compared with surgery

The major concern about thermal ablation is the high incidence of local recurrence and extrahepatic recurrence [29]. As a result, it would be anticipated that it will not offer similar cancer-related outcomes as liver resection surgery [29]. However, in patients who are at high surgical risk but would currently be considered for liver resection [30], the short- and long-term outcomes after surgery are poorer than the normal surgical cohort [9–14] and hence thermal ablation may be a valid alternative to surgery for this group.

### Uncertainty in treatment of patients with potentially resectable colorectal liver metastases

The HTA review suggested that there is equipoise regarding treatment using ablation or surgery of elderly patients and those with significant co-morbidities which pose significant risk from a surgical procedure and that good quality evidence of both the clinical benefit and cost-effectiveness of ablation is required [25]. In summary, liver resection is a modality with higher short-term mortality and complication rates and poorer quality of life during the first 3–6 months but with low local recurrence rates and a high potential for cure: > 25% of patients are alive five years after liver resection of CLM [31]; ablation is a less expensive modality with practically no short-term mortality, lower complication rates, earlier recovery, and higher short-term quality of life but with high local recurrence rates and the crucial uncertainty of whether it could offer similar long-term cancer-related outcomes as liver resection [30]. From a practical aspect, the choice in the treatment of patients with potentially resectable CLM lies between these two modalities.

### When can one consider ablation to be equivalent to liver resection in terms of long-term results?

Liver resection for CLM is a major surgical procedure and carries a postoperative mortality of approximately 3–4% and a complication rate of ~ 40% [31]. Because of the effects of major surgery and the associated pain, the patients take about 2–3 months to recover from surgery and the quality of life is only 0.65 on a scale of 0 to 1 (1 indicating perfect health) even six months after liver resection [25]. As the patient group identified for this trial are those considered high-risk in terms of age, co-morbidity and the extent of liver resection required, the mortality, morbidity and length of hospital stay are likely to be considerably higher than the average and the health-related quality of life and recovery period significantly greater.

In contrast, the ablative methods have fewer complications (6%) [26] and the quality of life is 0.74 (on a scale of 0 to 1) by three months [25]. An informal discussion with patient representatives from Bowel Cancer UK suggested they were willing to trade-off 3–6 months (average 4 months) of their long-term survival in return for a less-invasive procedure with significantly lower complication rates compared to surgery. So, on average, the long-term results of ablation and surgery can be considered equivalent if the difference in long-term survival between the modalities is < 4 months. Clearly, if ablation results in similar or better survival, ablation is the better option since it is less invasive and results in better quality of life in the short-term.

### Choice of patient group to be investigated

There has been no adequately powered RCT comparing ablation vs surgery in patients with CLM. Retrospective cohort studies highlight the high local recurrence rate associated with thermal ablation in comparison to liver resection surgery but a much lower procedure-related morbidity and mortality [21, 22]. The options include performing a RCT for low-risk patients (young patients without co-morbidities with limited extent of cancer spread) with CLM, high-risk patients with CLM, or all patients with CLM who are suitable for undergoing liver resection. There is good long-term data on the efficacy of surgical resection. Surgical resection provides low rates of local recurrence and disease-free survival (DFS) proportions of ~ 28% [31]. However, there is a high rate of local recurrence following thermal ablation and a lack of long-term data on the efficacy of ablation in patients with CLM. Because of the known rates of local recurrence and the lack of long-term data on cancer outcomes, the majority of clinicians feel that it is unethical to randomise low-risk patients to ablation or surgery despite the short-term benefits of lower complication rates, less pain and lower costs in patients undergoing ablation.

While some non-randomised studies did not justify these concerns and demonstrated equivalent survival between RFA and liver resection despite patients undergoing RFA having more co-morbidities or more extensive cancer [22, 26], another non-randomised study supported these ethical concerns and demonstrated that RFA had poorer five-year survival compared to surgery, the only difference between the patient groups in the second study being their preference for RFA or surgery [28].

However, with high-risk patients, there is significant uncertainty as to the benefits of surgery and the majority of clinicians feel that there is equipoise between these modalities for this patient group. These patients have 1.5–2 times lower survival than low surgical risk patients [12–14]. In this research, we will compare the effectiveness and cost-effectiveness of ablation vs surgery in this high-risk group of patients. If this research shows equivalent results of thermal ablation and surgery in this group, this will provide justification for a subsequent clinical trial on low-risk patients.

### Aim

The aim of this research is to compare the effectiveness and cost-effectiveness of thermal ablation vs liver resection surgery in high-risk patients with CLM.

## Methods

The trial has been registered in ISRCTN and can be accessed at http://www.isrctn.com/ISRCTN52040363. The SPIRIT (Standard Protocol Items: Recommendations for Interventional Trials) for this research is available in Additional file 1 [32].

### Design

A prospective, multicentre, open, pragmatic parallel randomised controlled non-inferiority trial design with internal pilot to investigate the effectiveness and cost-effectiveness of ablation (RFA or MWA) compared to liver resection for the treatment of patients with resectable CLM who would be considered high-risk for surgery and with a low chance of cure. The internal pilot will provide information about the feasibility of recruitment with a qualitative sub-study exploring the reasons why potential participants agree or do not agree to take part in the trial and methods of communication about the trial. The standard of surgical resections and thermal ablation procedures will be monitored during the internal pilot, along with assessment of data quality for key fields. Findings from the pilot will inform whether any changes are required to the trial design.

### Setting/context

Participating sites will be tertiary liver, pancreatic and gallbladder (HPB) centres. The trial will open in at least 20 research sites throughout the UK and the Netherlands. It is anticipated that at least four patients per site per year will be randomised into the trial, allowing the sample size of 330 patients to be recruited over a four-year period. In the 12-month pilot, we would expect at least 45 patients to be randomised with at least 15 sites open to recruitment to establish the feasibility of recruitment required for the main trial.

### Eligibility criteria for surgeons and radiologists

All surgeons and radiologists involved in the trial will have prior experience of performing relevant (resection or ablation) procedures for a minimum of 20 patients with liver cancer. The minimum standards to be achieved during the trial were discussed in the pre-trial standardisation meetings. Ablation therapy during open or laparoscopic surgery could be performed by either a radiologist or surgeon with sufficient experience.

### Sample size calculations

A total of 330 patients are required to demonstrate non-inferiority (non-inferiority margin of four months) of thermal ablation with respect to resection in terms of DFS with 80% power at the 2.5% one-sided level of significance, allowing for a four-year accrual period, two-year follow-up, and assuming 14 months DFS in the resection arm and allowing for a 5% drop-out rate.

### Participants

The trial will recruit 330 high surgical risk patients eligible for liver resection (165 in each arm). High-risk patients are defined as any patients that meet at least one of the following criteria: considered high-risk due to age (e.g. > 75 years); major co-morbidities as judged by the treating clinician; liver metastases with poor prognosis or high-risk surgery due to the tumour burden.

Patients will be identified for trial eligibility at the specialist liver multi-disciplinary treatment (sMDT) meeting. Suitability for inclusion into the trial will be assessed according to the following eligibility criteria.

#### Inclusion/exclusion criteria

Inclusion criteria

- Age ≥ 18 years
- Able to provide written informed consent
- MDT diagnosis of colorectal liver metastases considered to be resectable or ablatable with curative intent
- Resected colorectal primary or plan for primary resection with curative intent
- Meets *one or more* of the following criteria:
  - Considered high-risk for surgery due to age (based on local policy)
  - Major co-morbidities as judged by the treating clinician. Examples include history of myocardial infarction, severe chronic airway disease, major cerebrovascular accidents (CVA), pulmonary embolism (PE)
  - Liver metastases with poor prognosis and high-risk surgery due to tumour burden
    Examples include extensive synchronous disease, need for two stage resection or ALPPS, small anticipated remnant liver volume, curable extra-hepatic disease, downstaged with chemotherapy, poor response after chemotherapy but still resectable or ablatable
- Suitable candidate for either liver resection surgery or thermal ablation as judged by the MDT
- Able and willing to comply with the terms of the protocol including health-related quality of life (HRQoL) questionnaires

Exclusion criteria

- Incurable extra-hepatic metastases
- Not a suitable candidate for liver resection surgery
- Not a suitable candidate for thermal ablation
- Concurrent malignant disease (except basal cell carcinoma)
- Patients who have undergone previous surgery or ablation for CLM
- Planned simultaneous resection of primary and liver metastases
- Pregnancy

All patients will provide written informed consent. Patients will then be randomised on a 1:1 basis to receive either thermal ablation or surgical resection. A computer-generated minimisation programme that incorporates a random element will be used to ensure treatment groups are well-balanced for the following participant characteristics:

- Research site
- Synchronous/metachronous disease
- Primary cancer in situ (Yes/No)
- High-risk for surgery due to age (Yes/No)
- Major co-morbidity (Yes/No)
- Poor prognosis or high-risk surgery due to tumour burden (Yes/No)
- Largest lesion size ≤ 5 cm (Yes/No) [13]
- Planned surgical resection
  - Open
  - Laparoscopic
- Planned ablative treatment
  - RFA
  - MWA

Blinding will not be performed.

### Pre-treatment investigations

Pre-treatment investigations and preparation will be as per institutional protocol but must include a baseline staging computed tomography (CT) scan of chest, abdomen and pelvis minimum and tumour markers (CEA as a minimum) within six weeks of the start of treatment.

### Definition of index disease

Index disease is defined as the disease distribution of CLM metastases at the time of the most recent sMDT review before the treatment commencing.

### Intervention: thermal ablation (RFA or MWA)

#### Definition of the intervention in the ablation arm

For patients in the ablation arm, the intervention is defined as the collection of ablation sessions conducted as treatment for the index disease (as defined above). Either RFA or MWA will be carried out according to the local availability of equipment and expertise. Ablation may also be performed at laparoscopic or open surgery if the percutaneous approach is contraindicated. Minimum standards for delivery of ablation to be achieved during the trial will be agreed by all centres in a pre-trial standardisation meeting.

#### End of the intervention in the ablation arm

End of trial treatment in the ablation arm is defined as the end of the final ablation session in the collection of ablation sessions conducted as treatment of the index disease.

### Control: surgery

#### Definition of the intervention in the surgical resection arm

For patients in the surgical arm, the intervention is defined as the collection of operations conducted as treatment for the index disease (as defined above). Liver resection will be carried out as per centre protocol. The majority of patients will have undergone resection of the primary cancer. Patients may be offered open or laparoscopic liver resection depending on site and stage of disease. In selected cases, the liver first approach may be considered. Procedures for patients with extensive metastatic disease will include two-stage liver resection, venous embolisation or the ALPPS procedure (Associated Liver Partition and Portal vein ligation for Staged hepatectomy). Minimum surgical outcomes to be achieved during the trial will be agreed by all centres in a pre-trial standardisation meeting.

#### End of the intervention in the surgical arm

End of trial treatment in the surgical resection arm is defined as the end of the final operation in the collection of operations conducted as treatment of the index disease.

### Post-treatment care

Participants will be reviewed in clinic at 3, 6, 12, 18 and 24 months post randomisation. Follow-up imaging investigations (CT scan chest, abdomen and pelvis minimum) and tumour markers will be performed following completion of successful treatment of the index disease until disease recurrence.

### Further treatments

Further chemotherapy will be offered to patients as per current practice. There is currently no evidence from RCTs that adjuvant chemotherapy after surgical resection of CLM improves overall survival [33]. However, adjuvant chemotherapy after surgical resection improves DFS [33] and may be used in many centres. Treatment of recurrent disease will depend on the site and extent of disease and will be decided following review at the sMDT review and following discussion of the treatment options with patients and their family. Surgery and ablation are potentially curative treatment options for recurrent CLM [34–36]. If surgery or ablation is not considered feasible, then palliative chemotherapy is usually administered. The median survival for non-resectable recurrent CLM is approximately 15 months [36].

### Participant-completed questionnaires

Participant-completed questionnaires measuring quality of life (EQ-5D, EORTC QLQ-C30, EORTC LMC21) will be completed in clinic at baseline (following consent but before randomisation) and at 3, 6, 12, 18 and 24 months post randomisation. Patient-completed questionnaires measuring health and social care resource use will be completed at 3, 6, 12, 18 and 24 months post randomisation.

### Longer-term follow-up

Five-year survival data will be obtained from the Office of National Statistics (ONS).

### CT scan central review

All CT scans performed to assess the outcome of the ablation intervention (e.g. baseline scans and scans typically carried out around 4–6 weeks after the last ablative session of treatment) will be centrally reviewed for participants in the thermal ablation arm during the pilot phase. The central review will facilitate quality assurance of local interpretation of the CT scan findings, e.g. the completeness/‘success’ of the treatment, the need for further sessions, etc. To ensure quality assurance following the pilot study, review of thermal ablation will be carried out by the trial interventional radiologists in a further 20% of the patients chosen at random.

To ensure quality assurance of the reporting of recurrence, which feeds into the primary endpoint, a subset of CT scans performed during the follow up period (e.g. baseline scans and scans at around 3, 6, 12, 18 and 24 months post randomisation) will be chosen at random for central review. The selection process will be stratified by centre and outcome (recurrence/no recurrence) so that the accuracy of reporting of both ‘recurrence’ and ‘no recurrence’ can be assessed across all participating centres.

### Trial organisation

The LAVA trial is funded by the NIHR HTA Programme (grant reference 13/153/04). The trial is sponsored by University College London (UCL). The Clinical Trials Research Unit (CTRU) at the University of Leeds are responsible for the coordination of the trial. Trial supervision will be established according to the principles of Good Clinical Practice (GCP) and in line with the NHS Research Governance Framework (RGF). This includes the establishment of a core Project Team, Trial Management Group (TMG), an independent Trial Steering Committee (TSC) and DMEC.

### Ethical considerations

The trial will be performed in accordance with the recommendations guiding physicians in biomedical research involving human subjects adopted by the 18th World Medical Assembly, Helsinki, Finland, 1964, amended at the 64th World Medical Association General Assembly, Fortaleza, Brazil, October 2013. Informed written consent will be obtained from the participants before randomisation into the trial. The right of a patient to refuse participation without giving reasons must be respected. The participant must remain free to withdraw at any time from the trial without giving reasons and without prejudicing his/her further treatment. The trial has obtained ethical approval in the UK and in the Netherlands.

### Endpoints

#### Primary endpoint

DFS at two years post randomisation.

DFS is defined as the time from randomisation to the first event, which is defined as any of the following:

- Local, regional or extra hepatic/systemic recurrence of disease
- Death (any cause)

The time-to-event for patients whose treatment fails will be set equal to 0. If, according to post-intervention assessment, the index disease is deemed to have not been successfully removed/eradicated, then the treatment will be classed as having ‘failed’. Local recurrence is defined as the detection of disease at the treatment site after successful trial intervention. Regional recurrence is defined as detection of disease in the liver—not related to the treatment site—after successful trial intervention.

Extra-hepatic/systemic recurrence is defined as detection of new disease at any site other than the liver after successful trial intervention. ‘New disease’ refers to any extra-hepatic/systemic disease which was not already detected before commencement of trial treatment. The date of recurrence is defined as the date of the relevant assessment which detected the recurrence.

#### Secondary outcomes

1. Overall survival, defined as time from randomisation to death (any cause), evaluated at two and five years
2. Local, regional and extra-hepatic/systemic recurrence of disease at two years post randomisation (as defined above)
3. DFS (measured from end of intervention) at two years post randomisation (as defined above)
4. Use of subsequent therapies within two years post randomisation after treatment failure, for example, use of additional radiofrequency ablation or surgery for recurrence in either arm
5. Health-related quality of life (EQ-5D, EORTC QLQ-C30, EORTC LMC21) [37–39] at baseline, and 3, 6, 12, 18 and 24 months post randomisation
6. Complications during treatment
7. Post-treatment complications using Clavien-Dindo classification system [40, 41]
8. Length of intensive therapy unit (ITU) and inpatient stay (length of hospital related to treatment or treatment-related complications) at 30 days and 3 months after completion of treatment

Outcomes related to health economics and qualitative research are reported under the health economics section and qualitative research.

### Trial schema

**Fig. 1:**
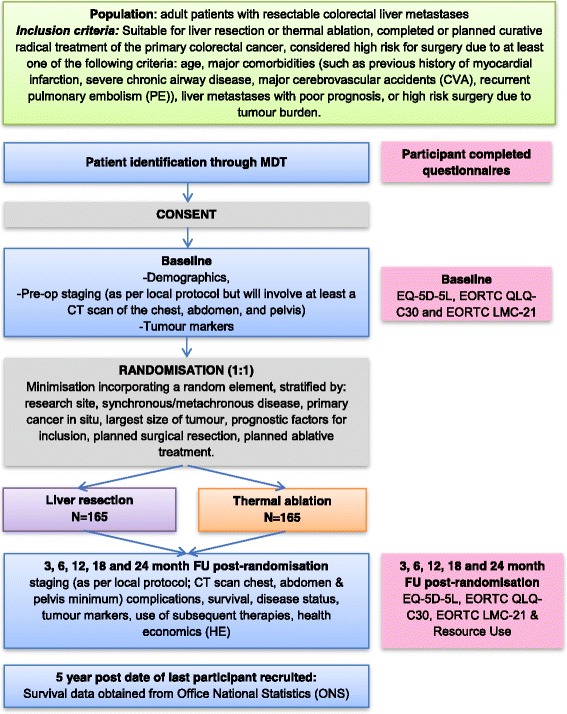
Trial schema. The figure shows the pathway that participants who are potentially eligible for the trial follow

The trial schema is shown in Fig. 1.

### Data analysis

#### Statistical analysis

Analysis and reporting will be in line with CONSORT guidelines. Analyses will be carried out on both an intention-to-treat (ITT) basis and per-protocol (PP) basis. Non-inferiority hypotheses will be tested at the one-sided 2.5% level of significance. Superiority hypotheses will be tested at the two-sided 5% level of significance. 95% confidence intervals for parameter estimates will also be reported.

The primary analysis will assess the difference in DFS. The non-inferiority hypothesis will be tested using an appropriate survival model to incorporate random effects with respect to research site and including adjustment for the stratification factors. The specific survival model that is most ‘appropriate’ cannot be determined a priori; for example, the Cox proportional hazards model will be considered but will not be used if the proportional hazards assumption is violated. Patients for whom an event is not reported during their trial follow-up will be censored at the last date that they were known to not have had an event.

Differences in rates such as complication rates and recurrence rates will be analysed using multi-level logistic regression incorporating random effects with respect to research site and will include adjustment for the stratification factors.

Subgroup analysis based on the type of surgery (open or laparoscopic liver resection) and type of ablation (RFA or MWA) will be performed. If there are truly substantial differences in efficacy between types of treatment within an arm, then these subgroup analyses, while likely having limited precision, will give an unbiased indication of the magnitude and direction of the differences. This will allow us to explore how the treatment effect partitions into these more precise components, which will allow us to assess the validity of assumption that there is no substantial difference in efficacy between open and laparoscopic surgery, and there is no substantial difference between RFA and MWA. This will also allow us to perform sensitivity analyses using imputation methods to assess the impact of changing the proportion of each type of treatment performed within arm on the primary treatment effect estimate. This will facilitate the generalisability of our inferences to wider practice and to potential changes in the uptake of each type of treatment over time.

Continuous measures such as length of ITU and hospital stay will be analysed using multi-level normal-errors regression incorporating random effects with respect to research site and including adjustment for the stratification factors. In the case of deviation from the Normality assumptions, the appropriately transformed variable will be analysed.

### Health economic analysis

We will undertake a detailed health economic analysis of the cost and cost-effectiveness of ablation vs liver resection for the treatment of high-risk patients with resectable CLM. The analysis can be used to evaluate whether ablation is good value for money in this patient group. Our analysis will conform to accepted economic evaluation methods [42]. The RCT is powered to show that ablation is non-inferior to surgical resection. There is clear evidence that cost-minimisation analyses in non-inferiority studies are inappropriate [43, 44]; therefore, we will undertake an incremental cost-effectiveness analysis. We will estimate cost-effectiveness during the ‘within-trial’ period (two years/within-trial analysis) and also over the expected lifetime of the patient (lifetime/long-run analysis). The within-trial analysis will be based on resource use, health-related quality of life and survival data collected in the trial; the long-run analysis will be based on a decision-analytical model constructed using trial data supplemented with data from published sources. In the within-trial analysis, quality-adjusted life-years (QALYs) will be calculated for each patient based on the survival data and health-related quality of life data collected during the trial. The latter will be based on the EQ-5D-5 L (www.euroqol.org), which will be collected at baseline and at 3, 6, 12, 18 and 24 months post randomisation. Patient-specific utility profiles will be constructed assuming a straight-line relation between each of the patient’s EQ-5D scores at each follow-up point. The QALYs experienced by each patient from baseline to two years will be calculated as the area underneath this profile. The within-trial cost analysis will be based on volume of resource use data collected for each patient during the trial. Costs will be measured from the NHS and personal social services (PSS) perspective. Cost components included in the analysis will consist of the detailed cost of the ablation procedures (including annuitised capital costs plus consumables), laparoscopic and open surgical resection procedures, the costs of treating the complications of these procedures, CT scans and other imaging tests, MDT meetings, costs of chemotherapy, contacts for receipt of chemotherapy, contacts and medications for treating the side effects of chemotherapy, plus other resource use associated with the cancer and its sequelae (e.g. outpatient attendances, hospital readmissions, palliative care, primary care contacts, prescribed medications, use of social services including hospice care). The volume of resource use for each cost component will be measured directly in the trial from patient records and using patient diaries; unit costs will be taken from standard published sources. Patient level resource use data will be multiplied by the unit costs and summed across all cost components to calculate total costs per patient over the two-year period.

Multiple imputation by chained equations will be used to deal with missing EQ-5D and resource use values. Subsequent analyses of imputed data will include variance correction factors to account for additional variability introduced into parameter values due to the imputation process.

Cost-effectiveness will be calculated as the mean cost difference between ablation and surgical resection divided by the mean difference in outcomes (DFS/QALYs) to give the incremental cost-effectiveness ratio (ICER). Non-parametric methods for calculating confidence intervals around the ICER based on bootstrapped estimates of the mean cost and QALY differences will be used [45]. The bootstrap replications will also be used to construct a cost-effectiveness acceptability curve, which will show the probability that use of ablative therapy is cost-effective at two years for different values of the NHS’ willingness to pay for an additional QALY, and a cost-effectiveness confidence ellipse. We will also subject the results to extensive deterministic (one-, two-way, multi-way, threshold) sensitivity analysis.

In the lifetime model, cost-effectiveness will be calculated in terms of the incremental cost per QALY gained. We will undertake a review of the Cost-Effectiveness Analysis (CEA) Registry (https://healtheconomics.tuftsmedicalcenter.org/cear4/home.aspx) and the NHS Economic Evaluation Database (NHS-EED, www.crd.york.ac.uk/) to identify previous economic models that might be adapted. We will then develop a new cost-effectiveness model that will be populated based on available evidence, including the data collected during the trial. Following decisions about model structure, a list of parameter estimates required for the model will be developed. The specific details of the data to be used to populate the model will be determined following the development of the structure and the systematic searches of the literature to identify existing models. We will undertake deterministic and probabilistic sensitivity analysis, the latter assuming appropriate distributions and parameter values [46]. As part of this, we will construct cost-effectiveness acceptability curves and cost-effectiveness confidence ellipses.

We will use the numerator of the ICER described above to calculate the budget impact of using ablation compared with surgical resection, multiplying the incremental cost (positive or negative) by the estimated eligible population size. We will also undertake a value-of-information study [46] to measure the maximum amount to money the NHS should be willing to pay for additional research to reduce uncertainty regarding the use of ablation vs surgical resection in this patient group.

### Qualitative sub-study

Recruitment to RCTs with very different treatment arms can be difficult and recruitment to surgical trials is particularly challenging [47]. Qualitative research can identify aspects of the trial design that hinder recruitment and identifying possible solutions [48, 49]. Studies show patient-related (difficulties of informed consent, understanding randomisation and preference for certain treatments) and clinician-related factors (concern about impact on the doctor–patient relationship, clinical equipoise, how trial is presented to patient) affect recruitment [48–51]. The current trial compares thermal ablation and surgery for liver metastases, so it is essential to understand and address barriers to recruitment in order to demonstrate our ability to undertake the main trial.

#### Aims

To qualitatively explore patient and clinician acceptability of the trial and recruitment processes to assist in optimisation of recruitment and follow-up strategies employed for the remainder of the trial.

#### Objectives

1. To qualitatively explore patients and clinicians’ acceptability of the trial to assist in optimisation of recruitment strategies employed for the definitive trial
2. Explore reasons for participation and non-participation of eligible patients
3. Understand patients’ experience of the randomisation process on decision-making
4. Understand why people refuse to participate or do not take up allocated treatment
5. Patient understanding of trial materials, i.e. do patients understand what will happen if they take part and do they understand what they are being randomised to
6. Acceptability of study procedures
7. Acceptability of randomisation
8. Explore clinical equipoise in the liver surgery community
9. Understand how information is presented to recruiters. In particular, explore the content and style of delivery and feed this back promptly to recruiters to improve practice

#### Method

##### Design: semi-structured interviews

A purposive sample of up to 20 patients will be recruited over nine months from across the pilot study sites, to include the three outcomes of consent: (1) participant consented and accepted treatment allocation; (2) participant consented to randomisation but refused the allocated treatment; (3) participant refused participation in the trial. In-depth, semi-structured interviews will explore patient perspectives of treatment, their understanding of the two treatments, reasons for taking part or refusing the trial and the acceptability of randomisation between the procedures.

A purposive sample of approximately 15 healthcare professionals (local principal investigators, recruiters) from across the pilot study sites will be interviewed. Participants will be selected on the basis of their ability to shed light on the recruitment process (initial discussion, recruiter interview). Interviews will explore their views about the trial, clinical equipoise and their understanding of the recruitment challenges.

Patient and staff interviews will be informed by a topic guide developed in conjunction with PPI representatives and informed by the literature. All interviews will be audio-recorded with permission.

Participant information sessions (recruiter encounters) will be audio-recorded (with permission) to examine how information is presented by recruiters and received and understood by patients, to identify issues potentially affecting trial recruitment. Information gleaned from the interviews and listening to the participant information sessions will inform the development of training materials for the main trial.

##### Data analysis

All interviews will be professionally transcribed verbatim and managed usingNVivo [52]. The data will be analysed using thematic analysis [53, 54] and coded independently by two researchers for emerging themes who will then compare codes and themes and resolve any disagreements by consensus. The analysis will be further refined by using constant comparison and contrastive approach, and looking for negative cases. A subset of up to 60 patient recruitment encounters (audio recordings of recruitment encounters) will be analysed using content analysis to identify potentially directive uses of language and good practice.

Information gained will be utilised to optimise patient information provided relating to the study (recruitment interview and PIS) and to advise clinical staff about how to describe the study, which will enable recruitment to be maximised in the main trial. Our own STAR trial [50] has confirmed the value of such approaches in improving recruitment. Emerging issues related to trial design and conduct that may be responsible for poor recruitment will be discussed with the trial team and a plan to improve recruitment during the pilot trial will be introduced if necessary. This may include reconsideration of eligibility criteria, redesign of study information, advice about presenting the study, discussions about equipoise or evidence, which may be addressed by changes to study information, protocol or training for recruiters.

## Discussion

With the development of new technologies, new methods for cancer treatment are being introduced into the healthcare market and need to be evaluated in terms of both efficacy and cost-effectiveness in comparison to competing therapies. Unless this is performed, newer, more cost-effective therapies will not be introduced into the NHS or costly treatments which are ineffective may be adopted. This problem applies at the present time with the recent introduction of thermal ablation techniques as an alternative to surgery for the treatment of patients with CLM. If effective, they should be more widely implemented in the NHS and the technology refined. If they are ineffective, support for more extensive surgery is required and the technology should be abandoned. It is important that any evaluation of effectiveness and cost-effectiveness between competing therapies should be based on RCTs, which ensure that similar types of people receive the two therapies. In addition, the inclusion criteria and exclusion criteria should be such that it should be possible to perform both therapies in an adequate way in order to maintain equipoise and to avoid a therapy being considered superior to another because of the selection of participants to be included in the trial. In the LAVA trial, we will include only participants who are eligible for ablation and surgery. Although we have left it for the local specialist MDT to make the decision as to whether the potential participant is eligible for both ablation and surgery, we anticipate that the participants included meet the CIRSE criteria for RFA for CLM, i.e. ≤ 5 lesions and tumour size does not exceed 3 cm at its longest axis [16].

Controversies in cost-effectiveness are frequently addressed through an NIHR-funded HTA. A recent report on clinical effectiveness and cost-effectiveness of ablative therapies in the management of liver metastases suggested that a trial investigating the effectiveness and cost-effectiveness of ablation vs surgery in patients with resectable CLM is necessary [25]. The long-term results of ablation and surgery can be considered equivalent only if the difference in long-term survival between the modalities is < 4 months due to the recovery period required following major liver surgery. However, the true difference in cancer survival between ablation and surgery in patients with CLM is not known.

‘NHS England Strategic and Operational Planning 2014 to 2019’ states the following ‘The healthcare system is facing the challenge of significant and enduring financial pressures. People’s need for services will continue to grow faster than funding, meaning that we have to innovate and transform the way we deliver high quality services, within the resources available, to ensure that patients, and their needs, are always put first.’ Clearly, it is important to maximise the health of people using the resources available.

This research will achieve this purpose of maximising the health of people using the resources available. The results of this research can be adopted immediately in the UK because of widespread availability of expertise in both thermal ablation techniques and liver resection surgery, resulting in maximisation of the health benefits using the resources available in a short period of time for high-risk patients with potentially resectable CLM. This research may also have indirect benefit for low surgical risk patients with CLM in the long term. The results of the current research will either justify the concerns of clinicians who consider that thermal ablation is inferior to surgery for the treatment of patients with CLM (in which case, no RCT comparing ablation and surgery will be conducted in low surgical risk patients as it is extremely unlikely that ablation is equivalent or better than surgery in low surgical risk patients if it offers worse results than surgery in high surgical risk patients) or it may reassure clinicians that ablation is an effective therapy in which case, a subsequent RCT may be conducted in low surgical risk patients.

## Additional file

Additional file 1: SPIRIT checklist. The completed SPIRIT (Standard Protocol Items: Recommendations for Interventional Trials) checklist for this protocol indicating the section in which the issue was covered is indicated in the table. (DOC 128 kb)
